# Dual Immunological Prognostic Models for Risk Stratification and Treatment Insights in Triple-Negative Breast Cancer

**DOI:** 10.3390/ijms27031494

**Published:** 2026-02-03

**Authors:** Shihua Lin, Hongjiu Wang, Zhenzhen Wang, Yuxuan Xiao, Menoudji Djetoyom Patrice, Li Wang, Xia Li, Yunpeng Zhang

**Affiliations:** 1 College of Bioinformatics Science and Technology, Harbin Medical University, Harbin 150081, China; lion_linshihua@163.com (S.L.); 15946080833@163.com (Y.X.); menoudjidjetoyom@gmail.com (M.D.P.); wangli@hrbmu.edu.cn (L.W.); 2 College of Biomedical Information and Engineering, Hainan Medical University, Haikou 571199, China; wanghongjiu@muhn.edu.cn (H.W.); wangzhenzhen@muhn.edu.cn (Z.W.)

**Keywords:** TNBC, single-cell, tumor immune microenvironment, prognostic model, drug prediction

## Abstract

Triple-negative breast cancer (TNBC) represents the most aggressive breast cancer subtype, with its highly heterogeneous tumor microenvironment posing substantial challenges for precision diagnosis and therapy. To address this, we aim to construct a novel prognostic framework based on tumor-immune interactions. Through integrative analysis of single-cell RNA sequencing data from 30 TNBC samples (106,132 cells), we identify key tumor expression metaprograms and uncover their interaction with an immunosuppressive dendritic-cell subset, a process associated with the NECTIN1–NECTIN4 axis. Leveraging these interactions, we developed and validated two immunological prognostic models using multi-cohort transcriptomic data, including the stress response tumor cell and pDC_CLEC4C prognostic model (SPSM) and the immune response tumor cell and pDC_CLEC4C prognostic model (IPSM). These models effectively stratified TNBC patients into distinct risk groups, with the low-risk group characterized by an immunologically active microenvironment and elevated expression of immune checkpoint genes, suggesting a potential responsiveness to immunotherapy. Furthermore, we identified several potential therapeutic agents, including imatinib and bortezomib. Collectively, our dual-model framework provides a tool for risk stratification, offers translational insights for precision treatment, and presents new directions for understanding TNBC heterogeneity and therapeutic development.

## 1. Introduction

Triple-negative breast cancer (TNBC) refers to a subtype of breast cancer negative for estrogen receptor (ER), progesterone receptor (PR), and human epidermal growth factor receptor 2 (HER2), accounting for 10–15% of all breast cancer cases [1]. Due to the lack of therapeutic targets, TNBC mainly relies on chemotherapy. However, it exhibits characteristics such as high heterogeneity, strong invasiveness, and poor prognosis, with a significantly higher five-year recurrence rate than other subtypes [2]. Single-cell transcriptome analysis has shown that the tumor microenvironment (TME) of TNBC possesses immunosuppressive features, including M2 macrophage enrichment and T cell exhaustion [3]. Therefore, in-depth deciphering of the molecular characteristics of TNBC and the regulatory mechanisms of the tumor microenvironment is of great significance for developing novel therapeutic strategies and improving patient prognosis.

Advances in single-cell sequencing technology have provided a powerful tool for dissecting the complexity of the TNBC TME and intercellular crosstalk. For instance, Shuning Ding et al. revealed the unique interaction pattern between T and B cells in TNBC through single-cell analysis, offering new insights for prognostic assessment and therapeutic target development [4]. The TME of TNBC is composed of various immune and stromal cells as well as their secreted factors, which collectively promote immune escape and disease progression [5]. Notably, TNBC is characterized by high tumor mutational burden (TMB) and PD-L1 expression, suggesting potential sensitivity to immune checkpoint inhibitors [6,7]. In recent years, multiple research teams have focused on developing TNBC prognostic models based on immune characteristics, such as the T cell-related model constructed by Siyu Guo et al. and the B cell signature gene model developed by Fangrui Zhao et al. [8,9]. While multigene signatures such as Oncotype DX have been widely adopted for prognostic evaluation in breast cancer, their utility and specificity for TNBC remain limited [10]. Furthermore, although tumor-immune interactions are known to influence tumor progression, prognostic models that explicitly leverage the crosstalk between functionally defined tumor cell states and specific immunosuppressive immune subsets are still lacking. To address this gap, we developed dual prognostic models based on interactions between defined tumor metaprograms and an immunosuppressive dendritic cell subset, offering a novel tool for TNBC risk stratification.

This study aims to systematically dissect the tumor microenvironment and epithelial cell heterogeneity of TNBC to identify key tumor metaprograms and elucidate their interaction mechanisms with immunosuppressive dendritic cell subsets. Based on these mechanisms, we will construct and validate a novel dual immunoprognostic model to enhance the precision of risk stratification for TNBC patients. Finally, integrating computational drug screening results, this study seeks to provide novel insights for the development of targeted therapeutic strategies.

## 2. Results

### 2.1. Construction of a Comprehensive Single-Cell Atlas for TNBC

**Figure 1:**
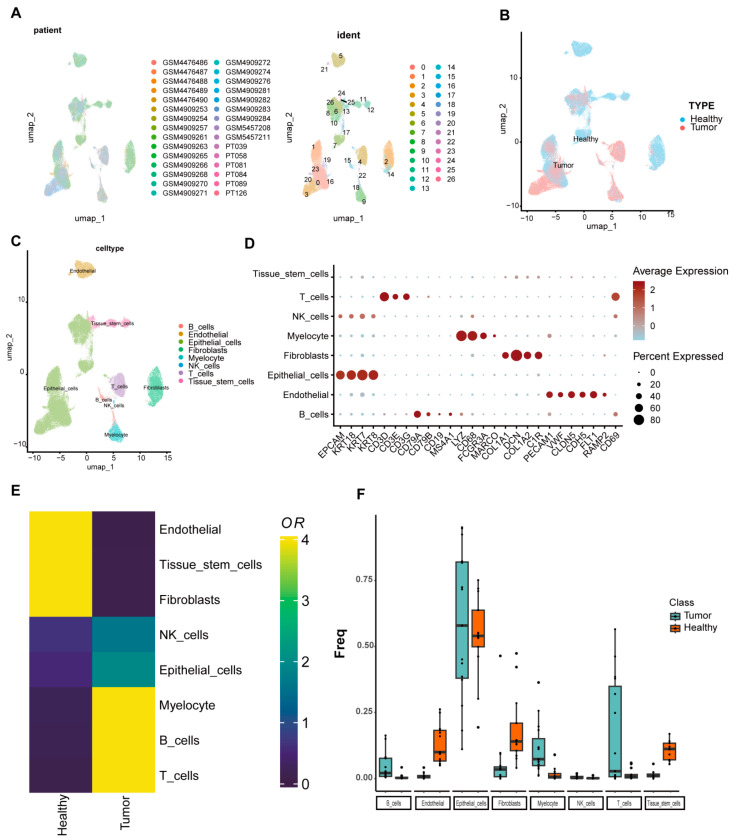
Construction of a comprehensive single-cell atlas of TNBC. (**A**) Integration and clustering of TNBC scRNA-seq data (17 normal, 13 tumor samples); (**B**) expression profiles of normal and tumor samples across clusters; (**C**) cell type annotation (eight types) using SingleR and marker gene signatures; (**D**) dot plot visualizing marker gene expression across eight cell types; (**E**) tissue distribution bias of cell types, quantified by odds ratio (OR); (**F**) box plot showing cell type proportions in normal versus tumor samples.

To construct a comprehensive single-cell atlas of TNBC, we analyzed scRNA-seq data from 17 normal and 13 cancer samples from GEO (Figure 1A). After quality control, 106,132 single-cell transcriptomes were retained and classified into eight cell types (endothelial, epithelial, fibroblasts, tissue stem cells, myeloid cells, NK cells, T cells, and B cells) using SingleR and marker gene analysis (Figure 1C,D). Normal samples showed higher proportions of endothelial cells, fibroblasts, and tissue stem cells, while cancer samples had more myeloid cells, T/B cells, and NK cells (Figure 1E). T cells emerged as the most abundant immune cell type in TNBC, consistent with Ding et al. [4] (Figure 1F).

### 2.2. Epithelial Cells in TNBC Exhibit Marked Intertumoral Heterogeneity

**Figure 2:**
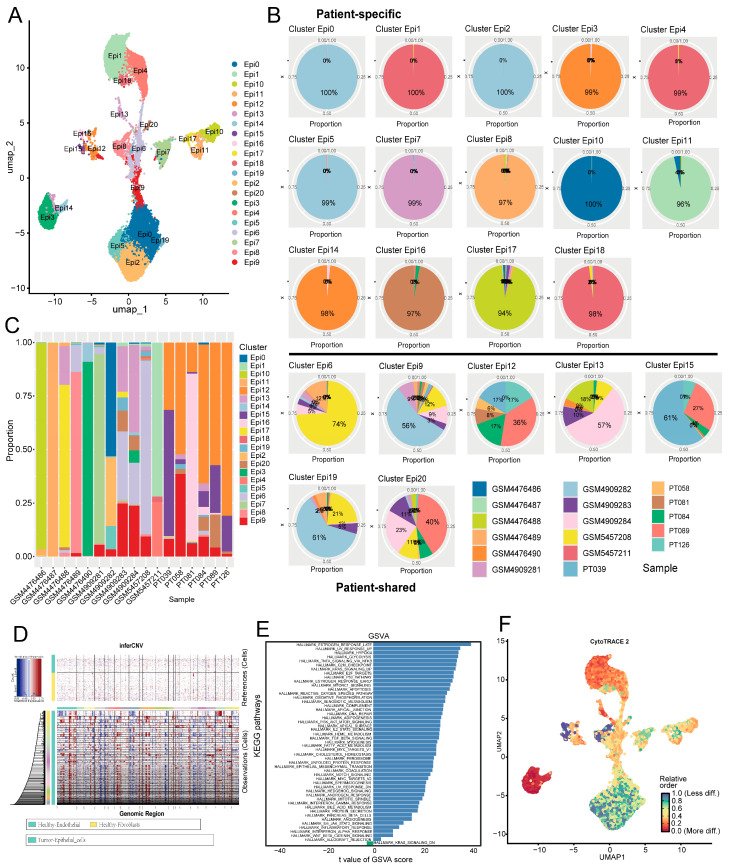
Analysis of intertumoral heterogeneity in TNBC, and identification of malignant epithelial cells through inferCNV analysis. (**A**) Visualization of epithelial clusters from 20 tumor samples; (**B**) tumor heterogeneity analysis: pie charts show sample contribution to subpopulations; (**C**) bar chart: subpopulation composition across tumor samples; (**D**) malignant epithelial cell identification by InferCNV; (**E**) GSVA pathway enrichment: patient-specific vs. patient-shared groups, blue and green represent the pathways enriched in the patient-specific group and the patient-shared group, respectively; (**F**) CytoTRACE2_Relative: Z-score scaled differentiation potential [0, 1].

To characterize epithelial cell expression states and dynamics during TNBC progression, we conducted dimensionality reduction and clustering on tumor-derived epithelial cells via the Seurat R package (v5.3.0), identifying 20 subclusters (Figure 2A). To objectively distinguish patient-specific from shared epithelial subpopulations, we analyzed the distribution of maximal patient contribution per cluster (Supplementary Figure S2). A distinct peak was observed at contribution levels ≥ 90%, while subclusters with lower contributions were broadly distributed. Using this 90% threshold, we defined patient-specific (maximum contribution ≥ 90%) and patient-shared subclusters. Accordingly, 14 subclusters (70%) were classified as patient-specific, with over 90% of cells derived from a single patient, while the remaining 6 were patient-shared (Figure 2B). This pattern indicates highly individualized epithelial transcriptomic profiles across TNBC patients. Further analysis showed that seven patients were predominantly patient-specific, six were mainly patient-shared, and four contained both types (Figure 2C). These results suggest substantial intertumoral heterogeneity in TNBC, aligning with Karaayvaz et al. [11], who reported TNBC subclonal heterogeneity and invasive states via scRNA-seq.

To clarify the malignant traits and dynamic changes in epithelial cells, we analyzed epithelial cells using the InferCNV algorithm, identifying malignant and non-malignant epithelial cells (Figure 2D). This analysis revealed clear differences in malignant cell proportions across epithelial subclusters (Supplementary Figure S3A), indicating distinct malignancy levels among subclusters. Notably, patient-specific group subclusters generally had higher malignant cell proportions than the patient-shared group. The Epi16 subcluster, for instance, showed marked malignant cell enrichment, further confirming the patient-specific group’s stronger malignant potential. Importantly, subcluster-specific differences in malignant cell proportions may correlate with tumor metastatic and invasive capacities. As prior studies [12] demonstrated, highly metastatic TNBC cells exhibit abnormally elevated metabolites in specific metabolic pathways and significantly upregulated key rate-limiting enzyme expression. This suggests distinct subclusters may harbor unique metabolic signatures that modulate metastatic and invasive potential.

Further pathway enrichment analysis demonstrated that estrogen late-response and hypoxia-related pathways were enriched in patient-specific group cells (Figure 2E). Despite TNBC lacking ER expression, late estrogen responses may regulate tumor cell growth and survival via non-canonical pathways, such as growth factor signaling and metabolic reprogramming [13]. Hypoxia, a key tumor microenvironment feature linked to malignant progression, was enriched in the patient-specific group. This implies these cells occupy a hypoxic niche and have adaptive low-oxygen survival mechanisms. For example, hypoxia can induce lncRNA GHET1 to activate the Hippo/YAP pathway, promoting TNBC cell glycolysis, proliferation, and invasion [14]. These findings indicate that cells in the patient-specific group possess stronger proliferative and invasive capabilities.

CytoTRACE2-based differentiation potential prediction for tumor epithelial subclusters showed Epi16 had the highest score, which suggests an early undifferentiated state, and Epi13 had the lowest score, which potentially represents a terminally differentiated stage (Figure 2F, Supplementary Figure S3B). Patient-specific subclusters also had markedly higher scores than patient-shared ones. This indicates less differentiation and greater malignancy, and the result is consistent with prior findings. In summary, the patient-specific group has a more aggressive malignant phenotype with strengthened proliferation and invasion capabilities.

### 2.3. Identification of Eight Common Expression Meta Programs in TNBC Malignant Cells

**Figure 3:**
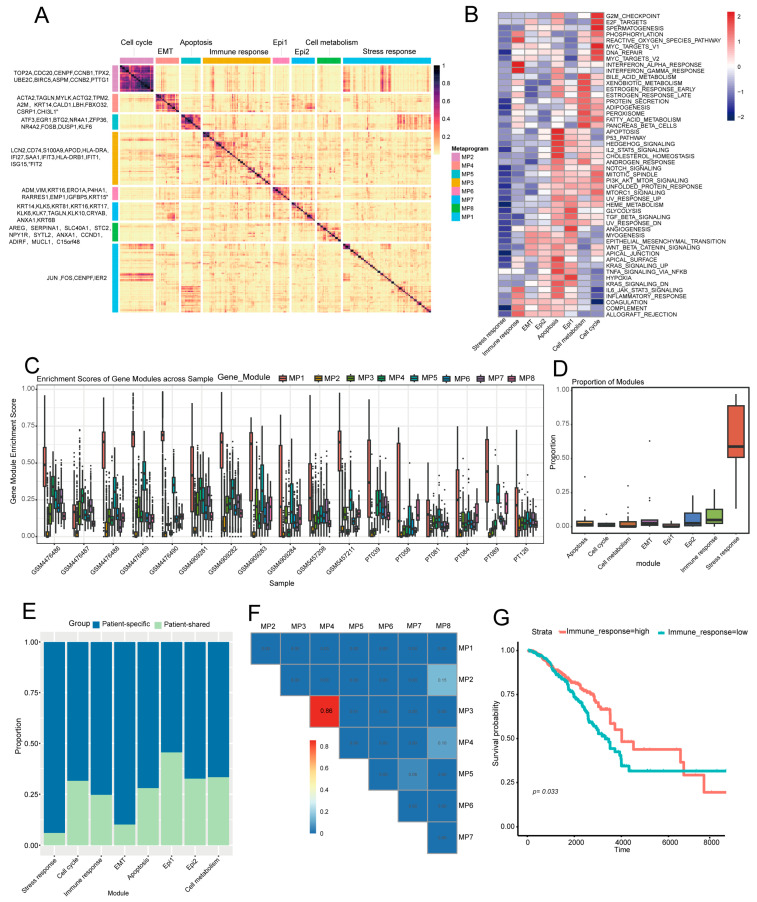
Identification of eight common expression metaprograms in TNBC malignant cells. (**A**) Heatmap showing the gene expression of each program in individual cells; (**B**) heatmap displaying the enrichment analysis of pathway activity in tumor cells for each meta-program; (**C**) enrichment analysis of gene meta-programs across samples; (**D**) box plot illustrating the proportion of cells corresponding to the eight meta-programs; (**E**) analysis of the proportion of cells from patient-specific and patient-shared groups within tumor cell subsets defined by meta-programs; (**F**) correlation analysis of the eight expressed meta-programs; (**G**) Survival analysis of the Immune_response meta-program.

We used Non-negative Matrix Factorization (NMF) to identify co-expressed metaprograms in malignant cells, ultimately defining eight distinct expression metaprograms with unique functions and cellular states (Figure 3A). We then applied the AddModuleScore algorithm to calculate each metaprogram’s enrichment score in individual cells and mapped each cell to the metaprogram with the highest score, which was designated as the corresponding “metaprogram cell”.

MP1 (stress response) includes core genes JUN, FOS, and IER2. JUN/FOS form the AP-1 complex, activated via pathways like MAPK under stress to regulate immune responses, proliferation, and differentiation [15]. IER2 fine-tunes stress responses and may modulate tumor progression [16] (Figure 3B). The synergistic action of these genes suggests MP1′s importance for cells to cope with environmental stress and maintain homeostasis. MP2 (cell cycle metaprogram) features high expression of proliferation-associated genes (TOP2A, CDC20, and CENPF), indicating active proliferation of its tumor cells. Functional enrichment analysis revealed enrichment in G2/M checkpoint, E2F targets, and MYC targets—pathways that collectively drive cell cycle progression and DNA replication. TOP2A regulates chromosome segregation/DNA topology [17], CDC20 is key to APC/C [18], and CENPF supports mitosis [19], suggesting MP2 tumor cells have high proliferative potential to promote tumor growth via sustained cell cycle progression. MP3 (Immune response metaprogram) has core immune-linked genes (LCN2, CD74, S100A9, and HLA-DRA) and activates pathways like inflammation and allograft rejection. LCN2/S100A9 regulates inflammatory signaling [20,21]; CD74/HLA-DRA supports antigen presentation [22]. This suggests MP3 tumor cells may face immune attack, though they could evade immunity via checkpoint upregulation [23]. MP4 (EMT metaprogram) is characterized by high expression of genes such as ACTA2, TAGLN, and MYLK, indicating tumor cells in this metaprogram are in an EMT state with aberrant Wnt/β-catenin pathway activation. Aberrant Wnt/β-catenin activation typically causes uncontrolled proliferation and accelerates tumor growth [24]. The EMT process enhances TNBC metastatic and invasive potential by boosting migration and invasion [25], so MP4 tumor cells exhibit active proliferation. MP5 (apoptosis metaprogram) includes genes such as ATF3, BTG2, and EGR1. Functional analysis showed its tumor cells have activated P53 signaling and cell death pathways, indicating possible apoptotic regulation—a host defense against DNA damage and tumorigenesis. P53 activation induces damaged cell apoptosis, reducing tumor initiation and progression [26]. MP6 (Epi1) features genes ADM, VIM, KRT16, and activated glycolysis, TGF-β signaling, and angiogenesis pathways. Glycolysis supplies tumor cell energy [27]. TGF-β signaling enhances invasion/migration via EMT [28]. Angiogenesis, induced by pathways like VEGF, forms new blood vessels for nutrient/oxygen supply [29]. These pathways collectively promote tumor growth, invasion, and metastasis. MP7 (Epi2) has epithelial differentiation/migration features with core genes KRT14, KLK5, and TAGLN and activated Notch, myogenesis, and EMT pathways. KRT14, a marker of epithelial differentiation [30], plays a key role in maintaining epithelial cell properties; the Notch signaling pathway is involved in tumor cell differentiation, stemness maintenance, and EMT processes [31]. These suggest Epi2 tumor cells are “partially differentiated,” retaining epithelial traits while gaining migration/invasion via these pathways, linked to tumor aggressiveness and metastasis. MP8 corresponds to the cell metabolism metaprogram, primarily composed of genes such as SLC40A1, C15orf48, and ELOVL5. Tumor cells in this metaprogram show activation of exogenous substance metabolism and peroxisome pathways. Peroxisomes regulate cellular immune signal transduction through redox metabolism, e.g., by participating in NF-κB activation. Additionally, peroxisomal β-oxidation and ether lipid synthesis play critical roles in the development and activation of innate and adaptive immune cells [32].

As observed, the cellular stress response metaprogram (MP1) shows high gene expression in most patients (Figure 3C) with a markedly high proportion of cells (Figure 3D). Over 90% of its cells derive from the patient-specific group and exhibit intense stress responses (Figure 3E). Under hypoxia and nutrient deprivation, tumor cells activate stress responses (e.g., oxidative stress and unfolded protein response) to promote survival, invasion, and metastasis [33]. This suggests that the TME in patients of the patient-specific group is more hostile. Furthermore, the EMT (MP4) and immune response (MP3) metaprograms have >70% of cells from the patient-specific group (Figure 3E) and show positively correlated synergistic dependency (Figure 3F), collectively driving tumor progression and metastasis. Studies confirm EMT enhances tumor migration/invasion and induces pro-tumorigenic TME changes; in breast cancer, elevated EMT markers (e.g., TWIST1, MMPs) associate with increased TME immune infiltration, which facilitates tumor immune evasion and accelerates progression/metastasis [34]. Survival analysis links the immune response metaprogram (MP3) to patient survival (*p* = 0.033, Figure 3G), highlighting its gene set’s critical role in TNBC progression and prognosis. Likely involved in immune activation, chemotaxis, and checkpoint regulation, this metaprogram is a potential TNBC prognostic biomarker; detecting its core gene expression may guide individual immunotherapy suitability.

### 2.4. The Interaction of pDC_CLEC4C with Stress Response and Immune Response Tumor Subpopulations Is Implicated in Immune Evasion

To investigate intercellular interactions within the TNBC tumor microenvironment, we performed unsupervised clustering analysis on major immune cell populations within the immune microenvironment, encompassing key immune components such as T cells, myeloid cells, and B cells.

**Figure 4:**
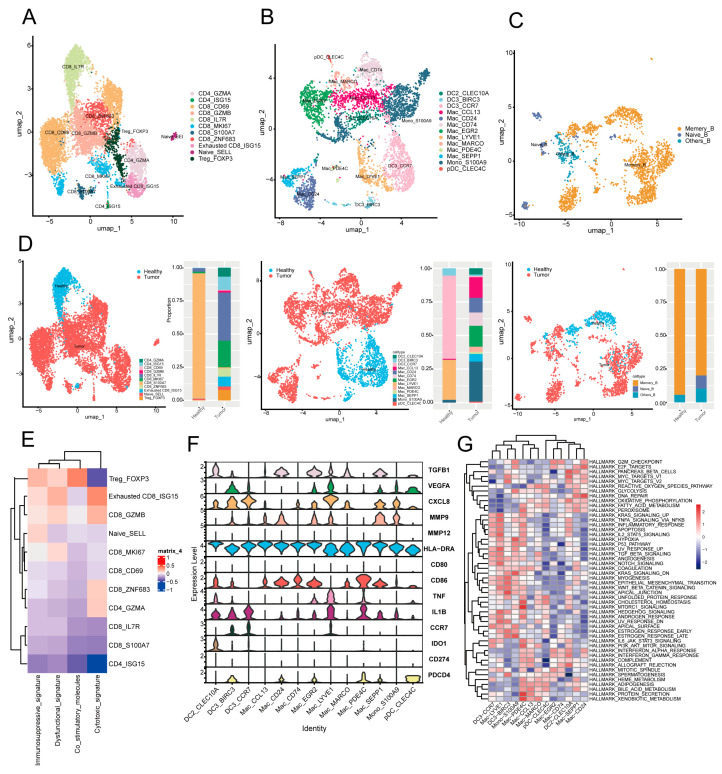
Functional characteristics of T cell, myeloid cell, and B cell subpopulations in the TNBC tumor microenvironment and their regulatory mechanisms in tumor progression. (**A**–**C**) UMAP plots of 11 T cell subpopulations (**A**), 13 myeloid cell subpopulations (**B**), and 4 B cell subpopulations (**C**) across all samples. (**D**) Bar plots show the proportions of various immune cell subpopulations in normal and cancer samples, with cells color-coded by subpopulation (top) and tissue (bottom). (**E**) Expression of immunosuppressive, dysfunctional, costimulatory molecule, and cytotoxic signatures in 11 T cell subpopulations. (**F**) Expression of characteristic functional genes in myeloid cells, different colors represent distinct genes. (**G**) GSVA pathway enrichment analysis of 13 myeloid cell subpopulations.

Previous studies confirm that T cells are the most abundant infiltrating immune cells in breast cancer TME. Unsupervised clustering of 7410 T cells identified 11 subsets (4 CD4+ and 7 CD8+ clusters; Figure 4A), including Naive_SELL (high CCR7/SELL expression) and Treg_FOXP3 (specific FOXP3/IL2RA expression), as well as Exhausted CD8_ISG15 (high PDCD1/LAG3/TIGIT expression). Signature gene enrichment analysis showed Treg_FOXP3 overexpresses immune checkpoint and co-stimulatory molecule-related genes (Figure 4E), indicating an immunosuppressive TNBC TME that may promote tumor immune evasion. Specifically, Tregs highly express surface immunosuppressive checkpoints (e.g., CTLA-4 and PD-1): CTLA-4 binds APC-derived B7 with higher affinity than CD28, blocking CD28-B7 signaling to inhibit T cell activation/proliferation; Treg co-stimulatory molecules (e.g., OX40) may enhance their immunosuppressive function; additionally, PD-1 binding to tumor/TME-derived PD-L1 suppresses T cell function, further facilitating tumor immune evasion [35].

Myeloid cells, critical in TME for immune suppression and angiogenesis [36], were re-clustered into 13 subsets: 8 macrophages, 1 monocyte, and 4 dendritic cell clusters (Figure 4B). Mac_EGR2, Mac_LYVE1, Mac_SEPP1 macrophages, and pDC_CLEC4C (plasmacytoid dendritic cells) highly express immunosuppressive (e.g., TGFB1) and angiogenic (e.g., VEGFA, CXCL8, and PDCD4) genes. This indicates pDC_CLEC4C exerts immunosuppression, potentially inhibiting T and NK cell activity to promote tumor immune evasion and an immunosuppressive microenvironment, consistent with prior studies [37]. GSVA showed pDCs are enriched in unfolded protein response, fatty acid metabolism, oxidative phosphorylation, and cholesterol homeostasis (Figure 4G). Unfolded protein response enrichment suggests pDCs may adapt to TME immune pressure via protein folding/stress regulation [38], playing a critical role in TNBC progression by modulating immunity and promoting tolerance—linked to poor prognosis, aligning with other research [39]. Three B cell subsets were identified (Figure 4C), with memory B cells markedly reduced in tumor patients (Figure 4D). This may relate to TME immunosuppression, e.g., T cell-mediated disruption of B cell regulation, impairing memory B cell function/survival [40].

**Figure 5:**
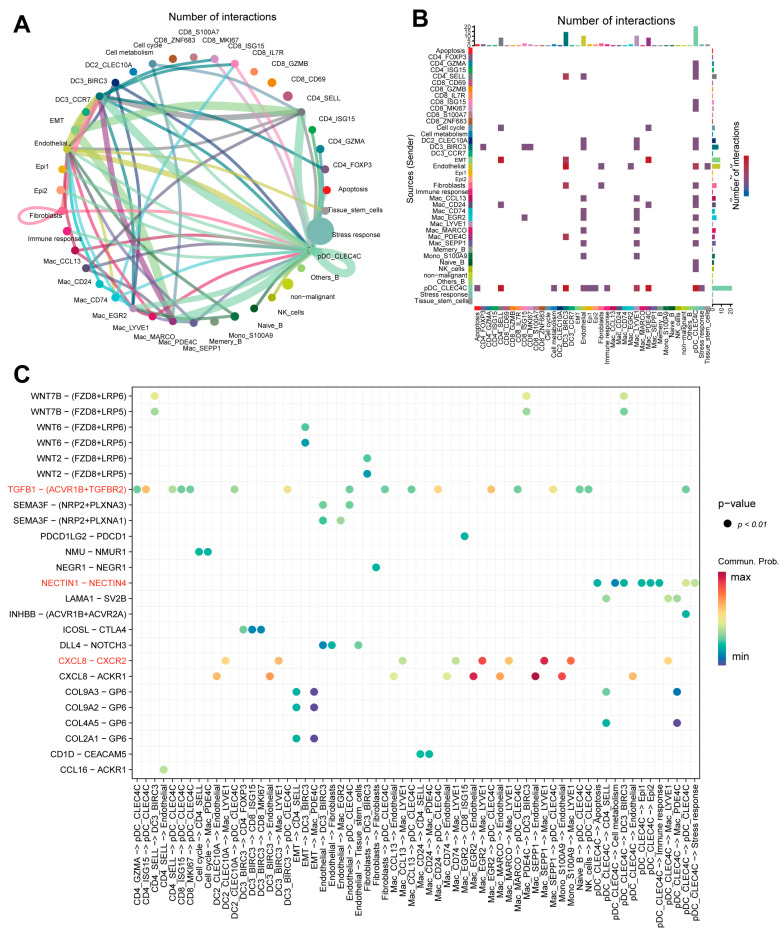
Dissection of complex interactions between immune cells and tumor cells in the TNBC tumor microenvironment. (**A**) Interactions between cell types and tumor cells in the TME: The size of colored circles in the periphery indicates the number of cells, where larger circles signify a higher cell count. Cells emitting arrows express ligands, while cells pointed to by arrows express receptors. Thicker lines denote a greater number of ligand–receptor pairs. (**B**) Heat map illustrating interactions between cell types and tumor cells in the TME. (**C**) Ligand–receptor pairs between cell types and tumor cells in the TME, where dot color represents the calculated communication probability and dot size indicates the *p*-value. Key ligand–receptor interaction pairs are highlighted in red.

CellChat analysis identified a complex intercellular interaction network in TNBC (Figure 5A,B). The pDC_CLEC4C immune subset interacted with multiple subsets, including tumor cell populations (apoptosis, cell metabolism, Epi1, Epi2, immune response, stress response) and others (e.g., CD4_SELL and endothelia). Since tumor-immune interactions are critical for tumor progression and therapy response [41], these data highlight TNBC TME complexity: tumor cells may regulate immune cell functions via secreted molecules (e.g., cytokines), weakening anti-tumor cytotoxicity to promote immune evasion and progression. Meanwhile, immune cells affect tumor cell growth and survival via secreted molecules (e.g., cytokines and antibodies) [42]. Tumor-immune interactions regulate tumor progression and contribute to immune evasion. For example, stress response/immune response tumor subsets and pDC_CLEC4C form NECTIN1-NECTIN4 ligand–receptor interactions (Figure 5C). NECTIN1/4 belong to the immunoglobulin superfamily and regulate cell junctions [43]; NECTIN4 is overexpressed in TNBC (barely detectable in normal breast tissue) [44], linked to breast cancer initiation, progression, invasion, and metastasis [45], and this interaction facilitates immune evasion, highlighting pDCs’ role in tumor immunity.

Additionally, COL9A3-GP6, COL9A2-GP6, and COL2A1-GP6 ligand–receptor interactions mediate crosstalk between CD4_SELL/Mac_PDE4C (immune subsets) and EMT tumor subsets. COL9A3/COL9A2/COL2A1 are collagen family members (key extracellular matrix (ECM) components) that regulate cell adhesion, migration, and tissue structure; aberrant collagen expression in the TME remodels the ECM to promote tumor proliferation and invasion [46]. Further analysis shows the TGFB1-(ACVR1B + TGFBR2) pathway is specifically activated in pDC_CLEC4C interactions with non-malignant cells (e.g., CD4_GZMA, endothelial cells). Since miR-425 inhibits TNBC progression by targeting TGF-β1 [47], this TGF-β1-related pathway may regulate TNBC immunity and progression. The Mac_LYVE1 subset interacts with other immune cell subsets (Mac_EGR2, Mac_SEPP1, and Mono_S100A9) via the CXCL8-CXCR2 ligand-receptor axis. Research indicates that CXCL8 and its cognate receptors CXCR1 and CXCR2 mediate the initiation and development of various cancers, including breast cancer [48], underscoring the significance of this axis in myeloid cell crosstalk within the TNBC TME.

### 2.5. Construction of the SPSM and IPSM Dual Immune Prognostic Models

**Figure 6:**
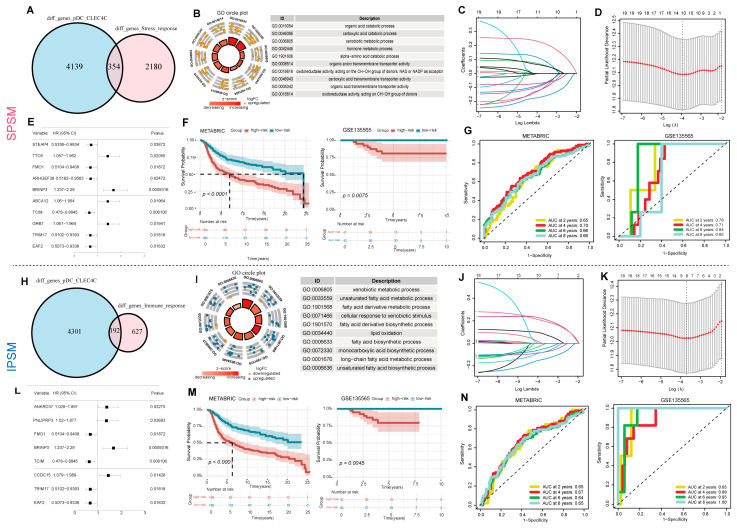
Construction of the SPSM and IPSM dual immune prognostic models. (**A**) The intersection in the Venn diagram represents the number of candidate genes associated with both Stress response metaprogram tumor cells and pDC_CLEC4C dendritic cells; (**B**) GO functional enrichment analysis of the candidate genes; (**C**) LASSO regression coefficient path plot. Each colored line represents a single variable in the prognostic model; (**D**) Partial likelihood deviance plot from LASSO regression. The red dotted curve represents the mean of the partial likelihood deviance derived from cross-validation, and the vertical dashed line indicates the λ value corresponding to the point of minimum deviance in the plot; (**E**) Forest plot of hazard ratios (HR) and 95% confidence intervals (CI) for each variable in the prognostic model. The black squares represent the point estimates of HR; (**F**) prognostic analysis of the training and testing cohorts with *p* < 0.05; (**G**) survival rates at 2, 4, 6, and 8 years for the SPSM in the training and testing cohorts; (**H**) the intersection in the Venn diagram represents the number of candidate genes associated with both immune response metaprogram tumor cells and pDC_CLEC4C dendritic cells; (**I**) GO functional enrichment analysis of the candidate genes; (**J**,**K**) construction of the prognostic risk model in the training cohort; (**L**) Forest plot showing the eight risk genes in the IPSM risk model; (**N**) prognostic analysis of the training and testing cohorts with *p* < 0.05; (**M**) Survival rates at 2, 4, 6, and 8 years for the IPSM model in the training and testing cohorts.

To screen key genes associated with stress response tumor cells and pDC_CLEC4C, we extracted differentially expressed genes in stress response tumor cells versus other tumor cells, then intersected them with differentially expressed genes in pDC_CLEC4C versus other myeloid cells, obtaining 354 candidates linked to both (Figure 6A). Functional enrichment analysis showed these genes were significantly enriched in metabolism-related processes, including organic acid and carboxylic acid catabolic processes (*p*.adjust < 0.05) (Figure 6B). Intersecting with TNBC microarray data and using univariate Cox regression (*p* < 0.05) yielded 20 prognosis-associated genes. LASSO and multivariate Cox regression further identified 10 key genes (STEAP4, TTC6, FMO1, ARHGEF38, BRINP3, ABCA12, TCIM, GRB7, TRIM17, and EAF2; Figure 6C,D), with their prognostic performance shown in a forest plot (Figure 6E). Based on these genes, we constructed a signature-based prognostic scoring model (SPSM) and subsequently stratified TNBC patients into low-risk and high-risk groups according to their calculated risk scores (Supplementary Note S1); survival analysis showed significantly poorer prognosis in high-risk patients in both training (METABRIC, *p* < 0.001) and test (GSE135565: *p* = 0.0075) sets (Figure 6F). Among these genes, GRB7 is associated with an increased risk of TNBC recurrence, and both GRB7 itself and GRB7-dependent signaling pathways may serve as potential therapeutic biomarkers. Concurrently, the STEAP family of metalloreductases, with a prominent role for STEAP4, is critical for cellular metal-ion homeostasis and represents a promising prognostic biomarker and novel therapeutic target in breast cancer [49,50]. To further validate the independent prognostic value of the risk model, we evaluated its predictive efficacy in TNBC patients using univariate and multivariate Cox regression analyses. Univariate analysis revealed that the risk score was a significant independent predictor of overall survival (HR = 1.54, 95% CI: 1.35–1.76, *p* < 0.001). In the multivariate analysis, the risk score retained its independent prognostic value (HR = 1.44, 95% CI: 1.21–1.72, *p* < 0.001) (Supplementary Figure S4A). Furthermore, the model exhibited high accuracy in predicting 2-, 4-, 6-, and 8-year survival rates in both the training and validation cohorts (AUC values: 0.65–0.84), further demonstrating its robust predictive capability (Figure 6G).

For the immune response meta-program, the same strategy was adopted to obtain 192 candidate genes (Figure 6H), which were enriched in functions such as xenobiotic metabolism and unsaturated fatty acid metabolism (Figure 6I). After screening, 8 key genes (including ANKRD37 and PNLIPRP3) were identified (Figure 6J,K), and a risk scoring model (IPSM) was constructed with the formula (Supplementary Note S2). The model significantly stratified high- and low-risk patients (METABRIC: *p* < 0.001; GSE135565: *p* = 0.0045; Figure 6M). The risk score was independently prognostic in both univariate (HR = 1.28, 95% CI: 1.17–1.41, *p* < 0.001) and multivariate analyses (HR = 1.15, 95% CI: 1.02–1.29, *p* = 0.019; Supplementary Figure S4B). It also consistently predicted 2-, 4-, 6-, and 8-year survival with AUC values of 0.64–1.00 across training and validation cohorts (Figure 6N). The two models shared five genes, namely FMO1, BRINP3, TCIM, TRIM17, and EAF2. Among them, TCIM (C8orf4) is an oncogene in breast cancer and is mechanistically associated with the FGFR signaling pathway [51].

### 2.6. The Low-Risk Group Is Characterized by High Infiltration of Multiple Immune Cell Types and Elevated Expression of Immune Checkpoints

**Figure 7:**
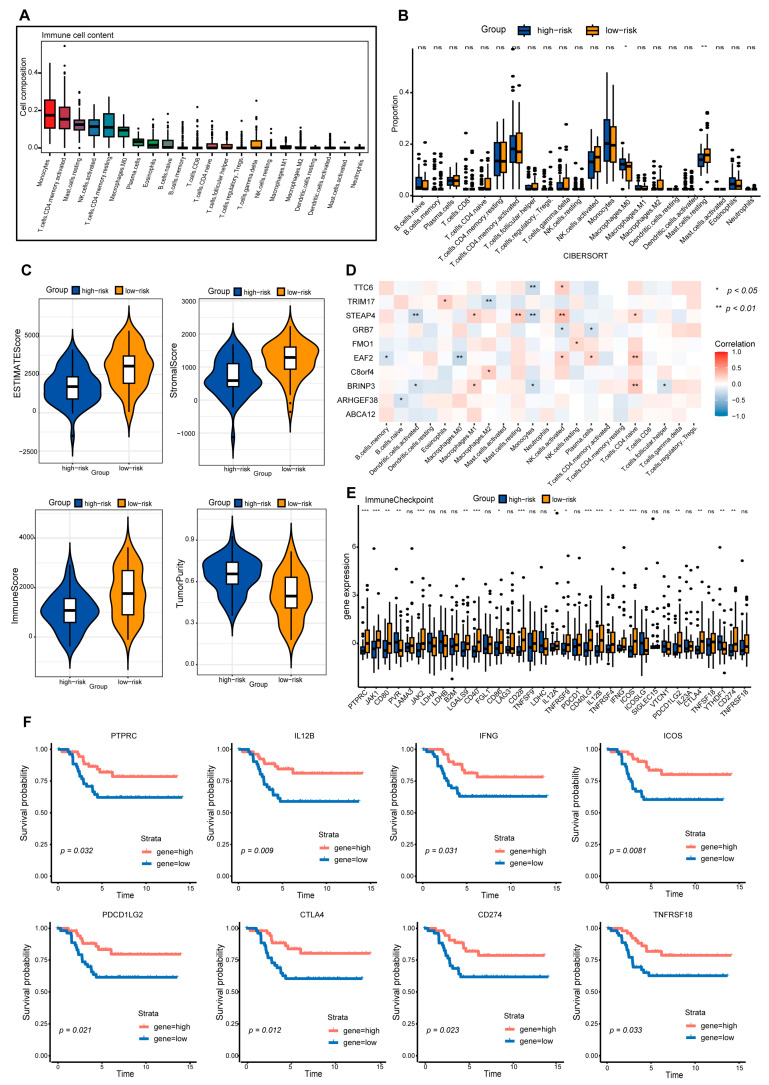
Differential analysis of immune cell infiltration in high-risk and low-risk TNBC patients based on the SPSM. (**A**) Immune infiltration profiles of 22 immune cell types in TNBC samples; (**B**) Differences in immune cell infiltration between the high-risk score group and the low-risk score group (** indicates *p* < 0.01, * indicates *p* < 0.05), “ns” indicates no significance. Blue represents the high-risk group, and yellow represents the low-risk group; (**C**) comparative analysis of stromal cell and immune cell score differences between high-risk and low-risk groups, the horizontal line inside the white box represents the median; (**D**) correlation between risk score genes and immune cell infiltration; (**E**) analysis of immune checkpoint expression levels between high-risk and low-risk groups (*** indicates *p* < 0.001, ** indicates *p* < 0.01, * indicates *p* < 0.05), “ns” indicates no significance; (**F**) immune checkpoints associated with TNBC prognosis.

In-depth dissection of immune cell composition/function in the TME is vital for advancing immunotherapy (Figure 7A and Figure S5A) [52]. This study systematically compared immune cell infiltration differences between high/low-risk TNBC groups defined by SPSM and IPSM models. Immune infiltration analyses from both models showed high-risk groups had higher T cells CD4 memory activated infiltration, while low-risk groups had higher infiltration of activated natural killer (NK) cells, M2 macrophages, and resting mast cells (Figure 7B and Figure S5B); further analysis revealed low-risk groups had higher immune scores and high-risk groups higher tumor purity (Figure 7C and Figure S5C), indicating low-risk TNBC patients had a more favorable immune status (Figure 7C) and providing a potential immunological basis for their better prognosis. Additionally, correlation analysis between risk score genes and immune cell infiltration showed most SPSM genes were negatively correlated with immune cells promoting immunotherapeutic responses (e.g., naive B cells, activated dendritic cells, plasma cells) and positively correlated with regulatory T (Treg) cell infiltration (Figure 7D); These genes collectively shape a tumor-permissive microenvironment conducive to immune evasion by promoting immunosuppressive Treg infiltration and inhibiting effector immune cell function. This process is closely associated with tumor progression and suggests a potential involvement in regulating immunotherapeutic sensitivity or resistance; these findings provide reference insights for exploring immunotherapy-related regulatory mechanisms, screening potential resistance biomarkers, and developing therapies targeting immunosuppressive pathways.

Differential analysis of immune checkpoint expression between high/low-risk groups showed low-risk patients (both models) had higher immune checkpoint gene expression than high-risk patients (Figure 7E and Figure S5E), with PTPRC, IL12B, IFNG, ICOS, PDCD1LG2, CTLA4, and CD274 closely associated with poor TNBC survival (Figure 7F). CTLA4 binds B7 molecules with higher affinity than CD28 to inhibit T cell activation/proliferation and negatively regulate immunity [53]; its high expression in TNBC may relate to immunosuppressive TME and correlates with poor prognosis [54]. CD274 (PD-L1) interacts with T cell PD-1 to suppress T cell function and facilitate tumor immune evasion, with its expression closely linked to TME immunosuppression in TNBC [55]; its genetic alterations and expression are extensively studied for associations with TNBC clinicopathological features and overall survival [56]. PTPRC mediates T/B cell activation signaling; IFNG exerts antiviral/antitumor effects, promotes TME immune cell infiltration and antitumor immunity by inducing tumor cell MHC/co-stimulatory molecule expression, and inhibits tumor angiogenesis/proliferation [56]. PDCD1LG2 (PD-L2) binds PD-1 to suppress T cell activation/proliferation and negatively regulate immunity; its high expression in TNBC may contribute to immunosuppressive TME and correlates with poor prognosis [57]. In summary, these immune checkpoint genes play critical roles in TNBC initiation, progression, and prognosis, with their expression closely linked to TME immune cell infiltration, antitumor immunity activation, or immunosuppression to influence clinical outcomes; further studies on their molecular mechanisms/regulatory networks will help unravel immune evasion mechanisms and provide a basis for developing novel immunotherapeutic targets and personalized strategies.

### 2.7. Combined Screening of Potential Targeted Drug Therapies for TNBC Using SPSM and IPSM

**Figure 8:**
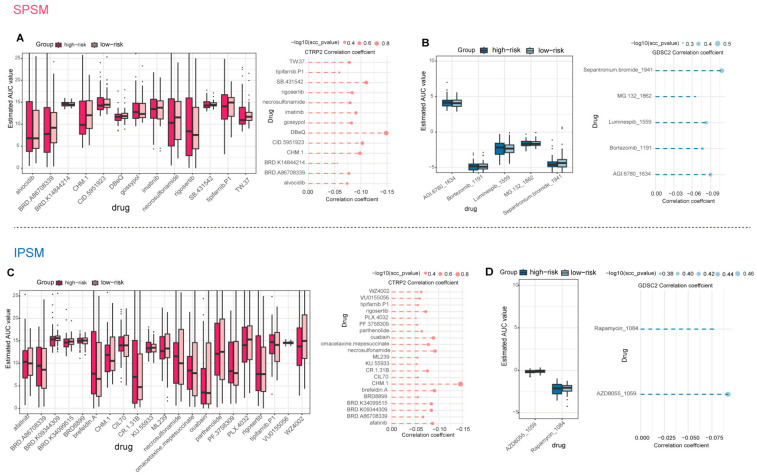
Prediction of potential therapeutic agents for TNBC. (**A**,**B**) Potential sensitive therapeutic agents predicted based on the risk genes of the SPSM. Among them, the dashed line represents the correlation coefficient value; (**C**,**D**) potential sensitive therapeutic agents predicted based on the risk genes of the IPSM model. Among them, the dashed line represents the correlation coefficient value.

We performed a systematic prediction of potential therapeutic agents for TNBC based on gene expression profiles and drug sensitivity data from the CTRP2 and GDSC2 databases. We set thresholds for the differential AUC values between high-risk and low-risk groups (Log_2_FC > 0) and for the negative correlation coefficients (r < −0.05) between the risk genes of the SPSM and IPSM models and AUC values, aiming to identify drugs with higher sensitivity in high-risk TNBC patients. These drugs exhibited lower AUC values in the high-risk group, indicating their potential for stronger therapeutic efficacy in high-risk TNBC patients. Through the aforementioned analysis, 13 potential drugs were identified from the CTRP2 database based on the risk genes of the SPSM (Figure 8A), including gossypol, SB.431542, DBeQ, CID.5951923, imatinib, BRD.K14844214, tipifarnib.P1, TW.37, CHM.1, rigosertib, necrosulfonamide, alvocidib, and BRD.A86708339. From the GDSC2 database, five potential drugs were screened out (Figure 8B), including Bortezomib_1191, Luminespib_1559, AGI.6780_1634, MG.132_1862, and Sepantronium. bromide_1941. Notably, imatinib and bortezomib have been clinically applied in the treatment of TNBC. Imatinib, classified as an antineoplastic agent, inhibits cancer cell growth, and previous studies support its potential as a novel therapeutic option for TNBC [58,59]. Bortezomib has also been utilized in the treatment of TNBC [60,61]. Meanwhile, three of the screened drugs, gossypol, rigosertib, and sepantronium bromide, have been previously confirmed as potential therapeutic agents for TNBC through preliminary studies. Gossypol effectively induces apoptosis in TNBC cells by specifically regulating the expression of apoptosis-related genes and exhibits enhanced sensitivity in TNBC cell lines derived from African Americans (MM-468), highlighting its potential value as a therapeutic agent for TNBC, particularly in African American female patients [62]. Furthermore, the natural polyphenol gossypol (GOSS), rigosertib, sepantronium bromide, and CHM-1 have been demonstrated as potential therapeutic agents for triple-negative breast cancer [63,64,65,66,67].

Based on the risk genes of the IPSM model, we screened 23 potential drugs, among which 21 were derived from the CTRP2 database: ouabain, KU.55933, CIL70, brefeldin A, parthenolide, tipifarnib.P1, CR.1.31B, afatinib, BRD8899, CHM.1, ML239, PLX.4032, BRD.K34099515, VU0155056, rigosertib, omacetaxine mepesuccinate, BRD.K09344309, necrosulfonamide, WZ4002, BRD.A86708339, and PF.3758309. The remaining two drugs were sourced from the GDSC2 database: AZD8055_1059 and Rapamycin_1084. Results showed that five drugs, namely tipifarnib.P1, CHM.1, rigosertib, necrosulfonamide, and BRD.A86708339, were commonly identified through the SPSM and IPSM models. Among these, rigosertib has been previously reported as a potential therapeutic agent for TNBC in prior studies [64]. Additionally, we identified five other drugs, including ouabain, parthenolide, afatinib, AZD8055, and rapamycin, that have been validated by previous research as potential therapeutic agents for TNBC [68,69,70,71,72,73,74]. KU.55933, brefeldin A, and PF-3758309 have been validated as potential therapeutic agents for breast cancer, and herein we propose their repurposing as candidate drugs for TNBC treatment [75,76,77].

## 3. Discussion

TNBC remains a highly challenging clinical problem due to its aggressive nature, pronounced heterogeneity, and limited treatment options. Recent studies have increasingly focused on deciphering the tumor microenvironment to identify prognostic biomarkers and therapeutic vulnerabilities, including T cell-related risk models and mitophagy-related prognostic models [8,78]. In this study, we integrated single-cell and bulk transcriptomic data to construct an immune prognostic evaluation model centered on tumor-immune interactions. This model not only stratifies TNBC patients into clinically relevant risk groups but also reveals potential immunosuppressive mechanisms and proposes candidate therapeutic agents.

Through integrative analysis of 30 TNBC single-cell RNA sequencing samples, we established a more detailed cellular atlas than previously published datasets [11]. Based on epithelial heterogeneity, TNBC epithelial cells were classified into patient-specific and patient-shared groups, revealing substantial inter-patient heterogeneity and a potential correlation with differential therapeutic responses, which may inform individualized TNBC treatment strategies. Eight tumor meta-programs (MP1-MP8) were identified via non-negative matrix factorization (NMF). Among them, epithelial–mesenchymal transition (MP4) and immune response (MP3) were coordinately enriched in specific patients, indicating their synergistic roles in promoting tumor invasion and immune escape [79]. Furthermore, this study revealed that pDC_CLEC4C dendritic cells are associated with immune suppression through the NECTIN1-NECTIN4 interaction, complementing previous mechanistic studies [80].

Based on the aforementioned findings, we developed two prognostic models, namely SPSM and IPSM, which effectively capture tumor-immune interaction features rather than relying solely on tumor-intrinsic genes. Compared with the model constructed by JingYing Li et al. [81], the models in this study more accurately reflect the interaction characteristics of the tumor-immune microenvironment. Compared with the study by Wang et al. [82], the models in this study provide a more detailed dissection of tumor-immune crosstalk and clarify the characteristics of potential immunotherapy sensitivity. Moreover, our models exhibit improved prognostic robustness: the SPSM achieved an AUC of 0.84 and the IPSM reached an AUC of 1.00 in validation analyses, reflecting their enhanced stability and discriminative power.

To bridge prognostic stratification with therapeutic translation, we employed a cross-database drug screening strategy, identifying both established drugs (imatinib, bortezomib) and novel candidate agents with predicted efficacy (tipifarnib and ouabain). This computational drug repurposing strategy aligns with recent research directions that link transcriptomic subtypes to drug sensitivity.

Despite these advances, our study has several limitations. The reliance on integrated public single-cell datasets introduces possible batch effects despite correction, and inferred cell–cell interactions require experimental validation. Additionally, drug predictions, though cross-validated computationally, necessitate preclinical and clinical confirmation. Future studies should further evaluate the clinical efficacy of the identified candidate drugs and experimentally validate the key genes in the prognostic models.

In conclusion, this study presents an integrative framework combining single-cell and bulk transcriptomics, linking tumor meta-programs, immune cell crosstalk, patient prognosis, and drug sensitivity. It provides critical insights into the pathogenesis, precise diagnosis, and treatment of TNBC, while also laying an important theoretical foundation for subsequent basic research and clinical interventions targeting TNBC.

## 4. Materials and Methods

### 4.1. Data Sources

We collected single-cell RNA-seq data from 13 normal and 17 tumor samples (GEO: GSE226391, GSE118389, GSE148673, GSE161529, GSE180286). Microarray data and corresponding clinical information of 299 TNBC patients from the METABRIC project were acquired from the cBioPortal database, while two independent TNBC microarray datasets with matched clinical data (GSE58812, *n* = 107; GSE135565, *n* = 84) were retrieved from the GEO database.

### 4.2. Cell Clustering and Cell Type Annotation

scRNA-seq data from TNBC samples were processed using Seurat (v5.3.0). Batch effects were corrected via Harmony (v1.2.3), and the batch-corrected expression matrix was used for further analyses (Supplementary Figure S1). After creating Seurat objects, QC was performed by removing low-quality cells (detected genes < 200 or mitochondrial RNA content > 20%) and genes expressed in <3 cells. Gene expression was normalized via LogNormalize (scale factor = 10,000). Highly variable genes were identified using FindVariableFeatures (vst method), retaining the top 2000 for downstream analysis. Dimensionality reduction was first conducted with PCA, retaining the top 30 PCs. Clustering resolutions from 0.1 to 1 were tested, and a resolution of 0.8 was ultimately selected for downstream analyses. UMAP dimensionality reduction and visualization were then performed using the top 30 principal components to display the cellular clustering structure. Cell types were annotated by combining SingleR (v2.4.0) predictions with canonical markers: epithelial (EPCAM, KRTs), endothelial (PECAM1, VWF, CDH5), fibroblasts (COL1A1, DCN), myeloid (LYZ, CD68), T cells (CD3D/E/G), B cells (CD79A/B, CD19), and NK cells (CD69).

### 4.3. Identification of Tumor Cells

To accurately identify malignant cell populations in TNBC epithelial cells, we inferred copy number variations (CNV) based on single-cell transcriptomic data. Specifically, we detected CNV aberrations by analyzing perturbation patterns in chromosomal gene expression levels, using non-epithelial cells (including immune and stromal cells) as normal reference controls. First, we preprocessed the data by filtering genes with low informativeness (defined as expressed in fewer than 10 cells or with an average expression below 0.1 on the log_2_ scale). Subsequently, expression values for each gene were converted to Z-scores and constrained within the range of −3 to 3 to normalize data distribution. Next, genes were sorted by their chromosomal positions, and a 100-gene sliding window was used to calculate moving averages, with data smoothed by centering across genes. Finally, cells were classified using two key parameters: (1) CNV signal strength (MS, mean square value) to estimate CNV signal intensity and (2) correlation (CORR) to calculate the correlation between each cell’s CNV and the average of cells with high CNV signals (top 5%). Cells were identified as malignant if their CNV signal strength (MS) > 0.02 or correlation (CORR) > 0.2.

### 4.4. Prediction of Epithelial Cell Differentiation States

We employed the CytoTRACE2 (v1.1.0) algorithm to infer the stemness and developmental potential of TNBC tumor epithelial cells. Developed by the Newman Lab, CytoTRACE2 is a computational method based on a deep learning framework whose model was trained and validated on single-cell RNA sequencing datasets from 28 tissue types across the entire developmental spectrum in humans and mice (31 datasets in total). This model classifies cells on a spectrum from terminally differentiated (differentiated: 0) to totipotent (totipotent: 1). We used normalized expression data of TNBC tumor epithelial cells and their subpopulation annotation data as inputs to run CytoTRACE2 for stemness inference. The algorithm performs the following steps: (1) data preprocessing: descendingly sort the gene expression values of each cell and make predictions based on the sorted rank; (2) cell developmental state prediction: load pre-trained model parameters to predict the developmental potential of cells; and (3) result post-processing: smooth the predicted values using diffusion methods and regularize them via k-nearest neighbor (kNN) and binning methods. Finally, we visualized the CytoTRACE2 prediction results, including potency_score_umap (UMAP showing cell potency scores), potency_category_umap (UMAP showing cell stemness classification), and rel_order_umap (UMAP showing the relative order of cell potency scores, ranging from 0 to 1). This analysis provides important insights for resolving the heterogeneity and developmental trajectories of TNBC tumor cells.

### 4.5. Analysis of Dependencies Among Meta Programs

We employed Pearson correlation analysis to investigate dependencies among tumor meta programs. The Pearson correlation coefficient, also referred to as the Pearson product-moment correlation coefficient, is a linear correlation metric and the most commonly used measure of association. Denoted as r, it quantifies the linear relationship between two variables, X and Y, with values ranging from −1 (perfect negative correlation) to 1 (perfect positive correlation). The magnitude of r reflects the strength of the linear association, where larger absolute values indicate stronger correlations.

### 4.6. Scoring of T Cell and Myeloid Cell Signature Gene Sets

We used the AddModuleScore function in Seurat to calculate scores for 11 T cell subset signatures (including immunosuppression, dysfunction, costimulatory molecules, and cytotoxicity) and 13 myeloid cell subset signatures. The AddModuleScore function computes a composite score for each cell by integrating the expression levels of genes within the signature sets, accounting for both individual gene expression and intergenic cooperative effects. Using Seurat’s visualization tools, we generated heatmaps to clearly display the distribution of these scores across different cell subsets, enabling straightforward comparison of score profiles between distinct immune cell populations.

### 4.7. Analysis of Communication Between Tumor Metaprogram Cells and Immune Cells

To investigate the intricate intercellular communication between tumor metaprogram cells and immune cell subsets, we employed the CellChat R package (v1.5.0). Cellular communication probabilities were computed using default parameters, and interactions involving cell groups with <10 cells were filtered out. Significance was assessed via permutation testing, with ligand–receptor pairs exhibiting a *p*-value < 0.05 considered statistically significant. The resulting communication networks were visualized using circle plots and heatmaps to illustrate the communication patterns between tumor and immune cell subsets.

### 4.8. Immune Cell Infiltration Analysis

In this study, we employed the CIBERSORT (v1.0.1) to quantitatively analyze immune cell infiltration in TNBC patients. Using the gene expression profiles from the GSE58812 dataset and the LM22 reference matrix (comprising 22 immune cell subtypes), we performed deconvolution via linear support vector regression to compute the proportions of immune cell populations in each sample, retaining only results with *p*-values < 0.05 to ensure reliability. TNBC patients were stratified into high- and low-risk groups based on prognostic risk scores. We further compared immune cell abundances between these groups using the Wilcoxon rank-sum test (*p* < 0.05) and visualized the heterogeneity of the immune microenvironment via stacked bar charts and heatmaps. Additionally, we analyzed the correlations between the expression levels of immune checkpoint genes (e.g., PD-1, PD-L1, and CTLA-4) and risk scores to uncover potential implications for immunotherapy.

### 4.9. Identification of Single-Cell Functional States via Integrative NMF and Pathway Enrichment Analysis

We applied non-negative matrix factorization (NMF) using the GeneNMF R package (v0.1.0) to malignant epithelial cells. Samples were split by origin, and multi-rank NMF (k = 4:9, nfeatures = 1000) was performed. Robust metaprograms (nMP = 8) were integrated using cosine similarity (weight.explained = 0.8, min.confidence = 0.3). For each cell, we computed enrichment scores for all MP gene sets using the AddModuleScore algorithm, enabling classification of cells into the highest-scoring MP category. Functional characterization of epithelial cell programs was performed using the 50 Hallmark gene sets from MSigDB [83]. We applied the GSVA R package (v2.0.0) to quantify pathway activity in an unsupervised manner, calculating enrichment scores to measure the activity of each Hallmark gene set across cell populations.

### 4.10. Construction and Validation of Prognostic Models

The METABRIC cohort served as the training set to construct the prognostic model, with the GSE135565 dataset used for independent validation. Differentially expressed genes (Benjamini–Hochberg adjusted *p*-value < 0.05 and an absolute log_2_ fold change (|logFC|) > 1) significantly associated with overall survival (OS) in TNBC were first identified, followed by univariate Cox regression (*p* < 0.05) to select candidate prognostic genes. To refine feature selection and prevent overfitting, LASSO regression was applied, and final gene contributions were determined by multivariate Cox regression. A risk score model was built using gene expression levels and regression coefficients, stratifying patients into high- and low-risk groups based on the median score. Model robustness was confirmed through external validation in the test cohort.

### 4.11. Prediction of Potentially Sensitive Drugs

To screen for potential targeted therapeutic agents, we utilized the pRRophetic R package (v0.5.0) [84] to predict drug responses by integrating gene expression profiles with drug sensitivity data from the CTRP2 and GDSC2 databases.

## Data Availability

All datasets utilized in this study were derived from public databases, with specific details as follows: Datasets GSE226391, GSE118389, GSE148673, GSE161529, and GSE180286 are deposited in the GEO database (https://www.ncbi.nlm.nih.gov/geo/ (accessed on 18 March 2025)); the TNBC microarray datasets GSE135565 and GSE58812, which include relevant clinical information, were also retrieved from this GEO database. Additionally, the microarray data and corresponding clinical information of the METABRIC project were obtained from the cBioPortal database (http://www.cbioportal.org/ (accessed on 26 March 2025)). All codes are available upon reasonable request.

## Supplementary Materials

The following supporting information can be downloaded at: https://www.mdpi.com/article/10.3390/ijms27031494/s1.

## References

[1] Wang W., Dong G., Yang Z., Li S., Li J., Wang L., Zhu Q., Wang Y. (2024). Single-cell analysis of tumor microenvironment and cell adhesion reveals that interleukin-1 beta promotes cancer cell proliferation in breast cancer. Anim. Models Exp. Med..

[2] Garrido-Castro A.C., Lin N.U., Polyak K. (2019). Insights into Molecular Classifications of Triple-Negative Breast Cancer: Improving Patient Selection for Treatment. Cancer Discov..

[3] Xu L., Li C. (2022). Single-Cell Transcriptome Analysis Reveals the M2 Macrophages and Exhausted T Cells and Intratumoral Heterogeneity in Triple-Negative Breast Cancer. Anti-Cancer Agents Med. Chem..

[4] Ding S., Qiao N., Zhu Q., Tong Y., Wang S., Chen X., Tian Q., Xiao Y., Shen K. (2023). Single-cell atlas reveals a distinct immune profile fostered by T cell-B cell crosstalk in triple negative breast cancer. Cancer Commun..

[5] Zheng H., Siddharth S., Parida S., Wu X., Sharma D. (2021). Tumor Microenvironment: Key Players in Triple Negative Breast Cancer Immunomodulation. Cancers.

[6] Schmid P., Adams S., Rugo H.S., Schneeweiss A., Barrios C.H., Iwata H., Diéras V., Hegg R., Im S.A., Shaw Wright G. (2018). Atezolizumab and Nab-Paclitaxel in Advanced Triple-Negative Breast Cancer. N. Engl. J. Med..

[7] Pardoll D.M. (2012). The blockade of immune checkpoints in cancer immunotherapy. Nat. Rev. Cancer.

[8] Guo S., Liu X., Zhang J., Huang Z., Ye P., Shi J., Stalin A., Wu C., Lu S., Zhang F. (2023). Integrated analysis of single-cell RNA-seq and bulk RNA-seq unravels T cell-related prognostic risk model and tumor immune microenvironment modulation in triple-negative breast cancer. Comput. Biol. Med..

[9] Zhao F., Zhao C., Xu T., Lan Y., Lin H., Wu X., Li X. (2023). Single-cell and bulk RNA sequencing analysis of B cell marker genes in TNBC TME landscape and immunotherapy. Front. Immunol..

[10] He M., Jiang Y.Z., Gong Y., Fan L., Liu X.Y., Liu Y., Tang L.C., Mo M., Hou Y.F., Di G.H. (2024). Intensive chemotherapy versus standard chemotherapy among patients with high risk, operable, triple negative breast cancer based on integrated mRNA-lncRNA signature (BCTOP-T-A01): Randomised, multicentre, phase 3 trial. BMJ.

[11] Karaayvaz M., Cristea S., Gillespie S.M., Patel A.P., Mylvaganam R., Luo C.C., Specht M.C., Bernstein B.E., Michor F., Ellisen L.W. (2018). Unravelling subclonal heterogeneity and aggressive disease states in TNBC through single-cell RNA-seq. Nat. Commun..

[12] Wang Z., Jiang Q., Dong C. (2020). Metabolic reprogramming in triple-negative breast cancer. Cancer Biol. Med..

[13] Zhu D., Zhao Z., Cui G., Chang S., Hu L., See Y.X., Lim M.G.L., Guo D., Chen X., Poudel B. (2018). Single-Cell Transcriptome Analysis Reveals Estrogen Signaling Coordinately Augments One-Carbon, Polyamine, and Purine Synthesis in Breast Cancer. Cell Rep..

[14] Wang Y., Liu S. (2021). LncRNA GHET1 Promotes Hypoxia-Induced Glycolysis, Proliferation, and Invasion in Triple-Negative Breast Cancer Through the Hippo/YAP Signaling Pathway. Front. Cell Dev. Biol..

[15] Eferl R., Wagner E.F. (2003). AP-1: A double-edged sword in tumorigenesis. Nat. Rev. Cancer.

[16] Wu W., Zhang X., Lv H., Liao Y., Zhang W., Cheng H., Deng Z., Shen J., Yuan Q., Zhang Y. (2015). Identification of immediate early response protein 2 as a regulator of angiogenesis through the modulation of endothelial cell motility and adhesion. Int. J. Mol. Med..

[17] Uusküla-Reimand L., Wilson M.D. (2022). Untangling the roles of TOP2A and TOP2B in transcription and cancer. Sci. Adv..

[18] Jeong S.M., Bui Q.T., Kwak M., Lee J.Y., Lee P.C. (2022). Targeting Cdc20 for cancer therapy. Biochim. Biophys. Acta Rev. Cancer.

[19] Liao H., Winkfein R.J., Mack G., Rattner J.B., Yen T.J. (1995). CENP-F is a protein of the nuclear matrix that assembles onto kinetochores at late G2 and is rapidly degraded after mitosis. J. Cell Biol..

[20] Jung B.K., Park Y., Yoon B., Bae J.S., Han S.W., Heo J.E., Kim D.E., Ryu K.Y. (2023). Reduced secretion of LCN2 (lipocalin 2) from reactive astrocytes through autophagic and proteasomal regulation alleviates inflammatory stress and neuronal damage. Autophagy.

[21] Chen Y., Ouyang Y., Li Z., Wang X., Ma J. (2023). S100A8 and S100A9 in Cancer. Biochim. Biophys. Acta Rev. Cancer.

[22] Boneva S.K., Wolf J., Rosmus D.D., Schlecht A., Prinz G., Laich Y., Boeck M., Zhang P., Hilgendorf I., Stahl A. (2020). Transcriptional Profiling Uncovers Human Hyalocytes as a Unique Innate Immune Cell Population. Front. Immunol..

[23] Yin S.S., Gao F.H. (2020). Molecular Mechanism of Tumor Cell Immune Escape Mediated by CD24/Siglec-10. Front. Immunol..

[24] Zhou Y., Xu J., Luo H., Meng X., Chen M., Zhu D. (2022). Wnt signaling pathway in cancer immunotherapy. Cancer Lett..

[25] Zheng C., Yan S., Lu L., Yao H., He G., Chen S., Li Y., Peng X., Cheng Z., Wu M. (2021). Lovastatin Inhibits EMT and Metastasis of Triple-Negative Breast Cancer Stem Cells Through Dysregulation of Cytoskeleton-Associated Proteins. Front. Oncol..

[26] Hernández Borrero L.J., El-Deiry W.S. (2021). Tumor suppressor p53: Biology, signaling pathways, and therapeutic targeting. Biochim. Biophys. Acta Rev. Cancer.

[27] Vander Heiden M.G., Cantley L.C., Thompson C.B. (2009). Understanding the Warburg effect: The metabolic requirements of cell proliferation. Science.

[28] Hao Y., Baker D., Ten Dijke P. (2019). TGF-β-Mediated Epithelial-Mesenchymal Transition and Cancer Metastasis. Int. J. Mol. Sci..

[29] Folkman J. (1971). Tumor angiogenesis: Therapeutic implications. N. Engl. J. Med..

[30] Van Keymeulen A., Rocha A.S., Ousset M., Beck B., Bouvencourt G., Rock J., Sharma N., Dekoninck S., Blanpain C. (2011). Distinct stem cells contribute to mammary gland development and maintenance. Nature.

[31] Takebe N., Miele L., Harris P.J., Jeong W., Bando H., Kahn M., Yang S.X., Ivy S.P. (2015). Targeting Notch, Hedgehog, and Wnt pathways in cancer stem cells: Clinical update. Nat. Rev. Clin. Oncol..

[32] Di Cara F., Savary S., Kovacs W.J., Kim P., Rachubinski R.A. (2023). The peroxisome: An up-and-coming organelle in immunometabolism. Trends Cell Biol..

[33] Luo J., Solimini N.L., Elledge S.J. (2009). Principles of cancer therapy: Oncogene and non-oncogene addiction. Cell.

[34] Singh S., Chakrabarti R. (2019). Consequences of EMT-Driven Changes in the Immune Microenvironment of Breast Cancer and Therapeutic Response of Cancer Cells. J. Clin. Med..

[35] Sato Y., Passerini L., Piening B.D., Uyeda M.J., Goodwin M., Gregori S., Snyder M.P., Bertaina A., Roncarolo M.G., Bacchetta R. (2020). Human-engineered Treg-like cells suppress FOXP3-deficient T cells but preserve adaptive immune responses in vivo. Clin. Transl. Immunol..

[36] Dabiri S., Huntsman D., Makretsov N., Cheang M., Gilks B., Bajdik C., Gelmon K., Chia S., Hayes M. (2004). The presence of stromal mast cells identifies a subset of invasive breast cancers with a favorable prognosis. Mod. Pathol..

[37] Knight A., Rihova L., Kralova R., Penka M., Adam Z., Pour L., Piskacek M., Hajek R. (2021). Plasmacytoid Dendritic Cells in Patients with MGUS and Multiple Myeloma. J. Clin. Med..

[38] Ramirez M.U., Hernandez S.R., Soto-Pantoja D.R., Cook K.L. (2019). Endoplasmic Reticulum Stress Pathway, the Unfolded Protein Response, Modulates Immune Function in the Tumor Microenvironment to Impact Tumor Progression and Therapeutic Response. Int. J. Mol. Sci..

[39] Sisirak V., Faget J., Gobert M., Goutagny N., Vey N., Treilleux I., Renaudineau S., Poyet G., Labidi-Galy S.I., Goddard-Leon S. (2012). Impaired IFN-α production by plasmacytoid dendritic cells favors regulatory T-cell expansion that may contribute to breast cancer progression. Cancer Res..

[40] Akama-Garren E.H., Yin X., Prestwood T.R., Ma M., Utz P.J., Carroll M.C. (2024). T cell help shapes B cell tolerance. Sci. Immunol..

[41] Pernot S., Evrard S., Khatib A.M. (2022). The Give-and-Take Interaction Between the Tumor Microenvironment and Immune Cells Regulating Tumor Progression and Repression. Front. Immunol..

[42] Binnewies M., Roberts E.W., Kersten K., Chan V., Fearon D.F., Merad M., Coussens L.M., Gabrilovich D.I., Ostrand-Rosenberg S., Hedrick C.C. (2018). Understanding the tumor immune microenvironment (TIME) for effective therapy. Nat. Med..

[43] Samanta D., Almo S.C. (2015). Nectin family of cell-adhesion molecules: Structural and molecular aspects of function and specificity. Cell. Mol. Life Sci..

[44] Zeindler J., Soysal S.D., Piscuoglio S., Ng C.K.Y., Mechera R., Isaak A., Weber W.P., Muenst S., Kurzeder C. (2019). Nectin-4 Expression Is an Independent Prognostic Biomarker and Associated with Better Survival in Triple-Negative Breast Cancer. Front. Med..

[45] Wang H., Sun D., Chen J., Li H., Chen L. (2023). Nectin-4 has emerged as a compelling target for breast cancer. Eur. J. Pharmacol..

[46] Provenzano P.P., Eliceiri K.W., Campbell J.M., Inman D.R., White J.G., Keely P.J. (2006). Collagen reorganization at the tumor-stromal interface facilitates local invasion. BMC Med..

[47] Liu Y., Chen J. (2018). miR-425 suppresses EMT and the development of TNBC (triple-negative breast cancer) by targeting the TGF-β 1/SMAD 3 signaling pathway. RSC Adv..

[48] Liu Q., Li A., Tian Y., Wu J.D., Liu Y., Li T., Chen Y., Han X., Wu K. (2016). The CXCL8-CXCR1/2 pathways in cancer. Cytokine Growth Factor Rev..

[49] Sparano J.A., Goldstein L.J., Childs B.H., Shak S., Brassard D., Badve S., Baehner F.L., Bugarini R., Rowley S., Perez E.A. (2011). Relationship between quantitative GRB7 RNA expression and recurrence after adjuvant anthracycline chemotherapy in triple-negative breast cancer. Clin. Cancer Res..

[50] Tian W., Chen Y., Ye T. (2024). Exploring the prognostic significance of STEAP2 and STEAP4 in various breast cancer subtypes. Asian J. Surg..

[51] Yang Z.Q., Moffa A.B., Haddad R., Streicher K.L., Ethier S.P. (2007). Transforming properties of TC-1 in human breast cancer: Interaction with FGFR2 and beta-catenin signaling pathways. Int. J. Cancer.

[52] Roma-Rodrigues C., Mendes R., Baptista P.V., Fernandes A.R. (2019). Targeting Tumor Microenvironment for Cancer Therapy. Int. J. Mol. Sci..

[53] Xu X., Dennett P., Zhang J., Sherrard A., Zhao Y., Masubuchi T., Bui J.D., Chen X., Hui E. (2023). CTLA4 depletes T cell endogenous and trogocytosed B7 ligands via cis-endocytosis. J. Exp. Med..

[54] Kothari C., Osseni M.A., Agbo L., Ouellette G., Déraspe M., Laviolette F., Corbeil J., Lambert J.P., Diorio C., Durocher F. (2020). Machine learning analysis identifies genes differentiating triple negative breast cancers. Sci. Rep..

[55] Adinew G.M., Messeha S., Taka E., Ahmed S.A., Soliman K.F.A. (2023). The Role of Apoptotic Genes and Protein-Protein Interactions in Triple-negative Breast Cancer. Cancer Genom. Proteom..

[56] Guo L., Li W., Zhu X., Ling Y., Qiu T., Dong L., Fang Y., Yang H., Ying J. (2016). PD-L1 expression and CD274 gene alteration in triple-negative breast cancer: Implication for prognostic biomarker. Springerplus.

[57] Mohamad Hanif E.A., Shah S.A. (2018). Overview on Epigenetic Re-programming: A Potential Therapeutic Intervention in Triple Negative Breast Cancers. Asian Pac. J. Cancer Prev..

[58] Blanchard Z., Mullins N., Ellipeddi P., Lage J.M., McKinney S., El-Etriby R., Zhang X., Isokpehi R., Hernandez B., Elshamy W.M. (2014). Geminin overexpression promotes imatinib sensitive breast cancer: A novel treatment approach for aggressive breast cancers, including a subset of triple negative. PLoS ONE.

[59] Alam M.S., Sultana A., Wang G., Haque Mollah M.N. (2022). Gene expression profile analysis to discover molecular signatures for early diagnosis and therapies of triple-negative breast cancer. Front. Mol. Biosci..

[60] Shen S., Du X.J., Liu J., Sun R., Zhu Y.H., Wang J. (2015). Delivery of bortezomib with nanoparticles for basal-like triple-negative breast cancer therapy. J. Control. Release.

[61] Tseng L.M., Liu C.Y., Chang K.C., Chu P.Y., Shiau C.W., Chen K.F. (2012). CIP2A is a target of bortezomib in human triple negative breast cancer cells. Breast Cancer Res..

[62] Messeha S.S., Zarmouh N.O., Mendonca P., Alwagdani H., Cotton C., Soliman K.F.A. (2019). Effects of gossypol on apoptosis-related gene expression in racially distinct triple-negative breast cancer cells. Oncol. Rep..

[63] Messeha S.S., Zarmouh N.O., Mendonca P., Cotton C., Soliman K.F.A. (2020). Molecular mechanism of gossypol mediating CCL2 and IL-8 attenuation in triple-negative breast cancer cells. Mol. Med. Rep..

[64] Liu Z., Mao S., Dai L., Huang R., Hu W., Yu C., Yang Y., Cao G., Huang X. (2024). Discovery of dual-targeted molecules based on Olaparib and Rigosertib for triple-negative breast cancer with wild-type BRCA. Bioorg. Med. Chem..

[65] Kaneko N., Yamanaka K., Kita A., Tabata K., Akabane T., Mori M. (2013). Synergistic antitumor activities of sepantronium bromide (YM155), a survivin suppressant, in combination with microtubule-targeting agents in triple-negative breast cancer cells. Biol. Pharm. Bull..

[66] Liu C.W., Lin Y.C., Hung C.M., Liu B.L., Kuo S.C., Ho C.T., Way T.D., Hung C.H. (2018). CHM-1, a novel microtubule-destabilizing agent exhibits antitumor activity via inducing the expression of SIRT2 in human breast cancer cells. Chem. Biol. Interact..

[67] Wani T.H., Chowdhury G., Chakrabarty A. (2021). Generation of reactive oxygen species is the primary mode of action and cause of survivin suppression by sepantronium bromide (YM155). RSC Med. Chem..

[68] López-Tejada A., Blaya-Cánovas J.L., Cara F.E., Calahorra J., Ramírez-Tortosa C., Blancas I., Delgado-Almenta V., Muñoz-Parra F., Ávalos-Moreno M., Sánchez A. (2025). Signature-based repurposed drugs resemble the inhibition of TGFβ-induced NDRG1 as potential therapeutics for triple-negative breast cancer. Int. J. Biol. Sci..

[69] Araújo T.G., Vecchi L., Lima P., Ferreira E.A., Campos I.M., Brandão D.C., Guimarães G.S., Ribeiro M.A., Filho A. (2020). Parthenolide and its Analogues: A New Potential Strategy for the Treatment of Triple-Negative Breast Tumors. Curr. Med. Chem..

[70] Pellecchia S., Franchini M., Viscido G., Arnese R., Gambardella G. (2024). Single cell lineage tracing reveals clonal dynamics of anti-EGFR therapy resistance in triple negative breast cancer. Genome Med..

[71] Canonici A., Browne A.L., Ibrahim M.F.K., Fanning K.P., Roche S., Conlon N.T., O’Neill F., Meiller J., Cremona M., Morgan C. (2020). Combined targeting EGFR and SRC as a potential novel therapeutic approach for the treatment of triple negative breast cancer. Ther. Adv. Med. Oncol..

[72] Li H., Liu L., Chang H., Zou Z., Xing D. (2018). Downregulation of MCL-1 and upregulation of PUMA using mTOR inhibitors enhance antitumor efficacy of BH3 mimetics in triple-negative breast cancer. Cell Death Dis..

[73] Ferrari P., Scatena C., Ghilli M., Bargagna I., Lorenzini G., Nicolini A. (2022). Molecular Mechanisms, Biomarkers and Emerging Therapies for Chemotherapy Resistant TNBC. Int. J. Mol. Sci..

[74] Lin P.H., Tseng L.M., Lee Y.H., Chen S.T., Yeh D.C., Dai M.S., Liu L.C., Wang M.Y., Lo C., Chang S. (2022). Neoadjuvant afatinib with paclitaxel for triple-negative breast cancer and the molecular characteristics in responders and non-responders. J. Formos. Med. Assoc..

[75] Aiyappa-Maudsley R., Elsalem L., Ibrahim A.I.M., Pors K., Martin S.G. (2022). In vitro radiosensitization of breast cancer with hypoxia-activated prodrugs. J. Cell. Mol. Med..

[76] Wang M., Sun B., Ye T., Wang Y., Hou Y., Wang S., Pan H., Hua H., Li D. (2023). 5-(4-Hydroxyphenyl)-3H-1,2-dithiole-3-thione derivatives of brefeldin A: Design, synthesis and cytotoxicity in MDA-MB-231 human breast cancer cells. Bioorg. Med. Chem..

[77] Deng H., Xiao B., Huang Y., Weng K., Chen J., Li K., Wu H., Luo S., Hao W. (2022). The Combined Use of Orf Virus and PAK4 Inhibitor Exerts Anti-tumor Effect in Breast Cancer. Front. Microbiol..

[78] Ding P., Pei S., Qu Z., Yang Y., Liu Q., Kong X., Wang Z., Wang J., Fang Y. (2024). Single-cell sequencing unveils mitophagy-related prognostic model for triple-negative breast cancer. Front. Immunol..

[79] Pastushenko I., Blanpain C. (2019). EMT Transition States during Tumor Progression and Metastasis. Trends Cell Biol..

[80] Costa A., Kieffer Y., Scholer-Dahirel A., Pelon F., Bourachot B., Cardon M., Sirven P., Magagna I., Fuhrmann L., Bernard C. (2018). Fibroblast Heterogeneity and Immunosuppressive Environment in Human Breast Cancer. Cancer Cell.

[81] Li J.Y., Hu C.J., Peng H., Chen E.Q. (2024). A novel immune-related long noncoding RNA (lncRNA) pair model to predict the prognosis of triple-negative breast cancer. Transl. Cancer Res..

[82] Wang X., Chen H. (2022). Prognosis Prediction Through an Integrated Analysis of Single-Cell and Bulk RNA-Sequencing Data in Triple-Negative Breast Cancer. Front. Genet..

[83] Liberzon A., Birger C., Thorvaldsdóttir H., Ghandi M., Mesirov J.P., Tamayo P. (2015). The Molecular Signatures Database (MSigDB) hallmark gene set collection. Cell Syst..

[84] Geeleher P., Cox N., Huang R.S. (2014). pRRophetic: An R package for prediction of clinical chemotherapeutic response from tumor gene expression levels. PLoS ONE.

