# Supplementary material for: Dual Immunological Prognostic Models for Risk Stratification and Treatment Insights in Triple-Negative Breast Cancer
Source: Int J Mol Sci. 2026 Feb 3;27(3):1494. doi: 10.3390/ijms27031494 (PMC12898472; doi:10.3390/ijms27031494)
Supplement: Supplementary file 1 [file ijms-27-01494-s001.zip › ijms-4108946-supplementary.pdf]

Supplementary Materials

Supplementary NoteS

Supplementary Note S1：SPSM risk score calculation
Risk score = (−0.12359 × STEAP4) + (0.06953 × TTC6) + (−0.17092 × FMO1) + (−0.09310 × ARHGEF38) + (0.22714 × BRINP3) + (0.11132 × ABCA12) + (−0.07027 × TCIM) + (0.06279 × GRB7) + (−0.08477 × TRIM17) + (−0.12077 × EAF2),
Coefficients were obtained from Cox regression.

Supplementary Note S2：IPSM risk score calculation
Risk score = (0.08374 × ANKRD37) + (−0.00891 × PNLIPRP3) + (−0.14186 × FMO1) + (0.21774 × BRINP3) + (−0.07945 × TCIM) + (0.19927 × CCDC15) + (−0.04859 × TRIM17) + (−0.07746 × EAF2),
Coefficients were obtained from Cox regression.

Supplementary Figures

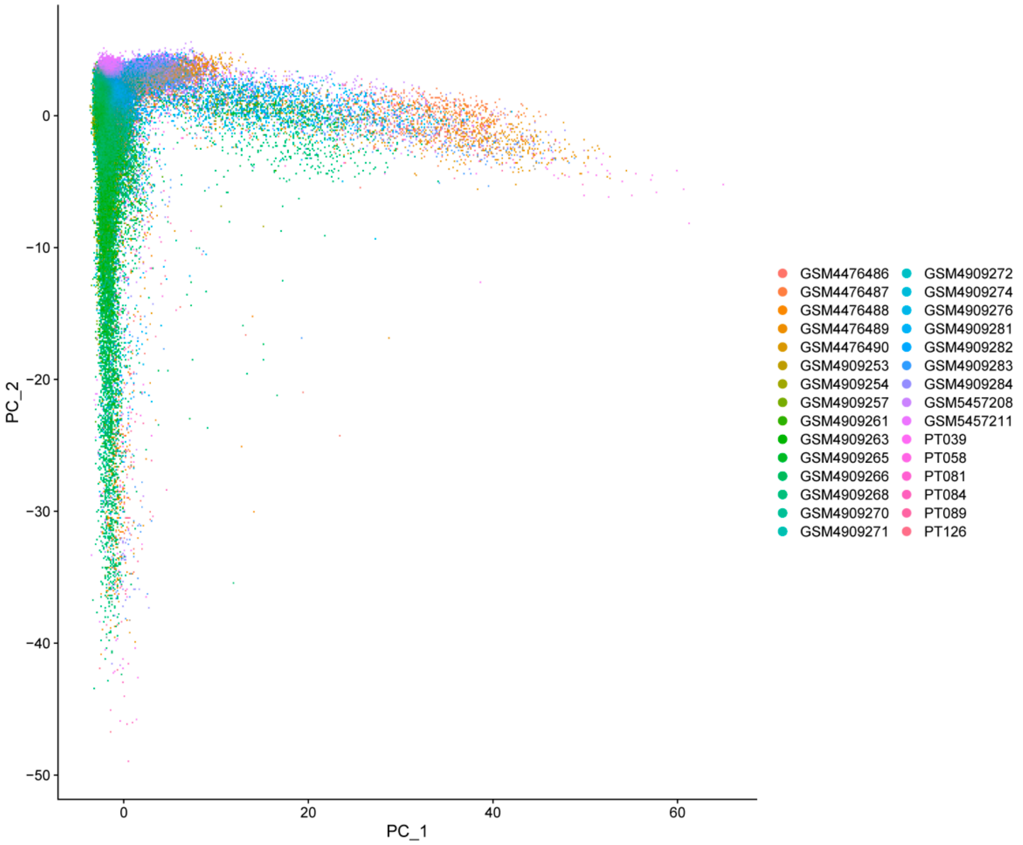

Supplementary Figure S1. Principal component analysis (PCA) of integrated single-cell TNBC data after Harmony-based batch correction.

The PCA plot visualizes the distribution of cells along the first two principal components (PC1 and PC2) following integration and batch correction using Harmony (v1.2.3). Each point represents a single cell and is colored by its sample.

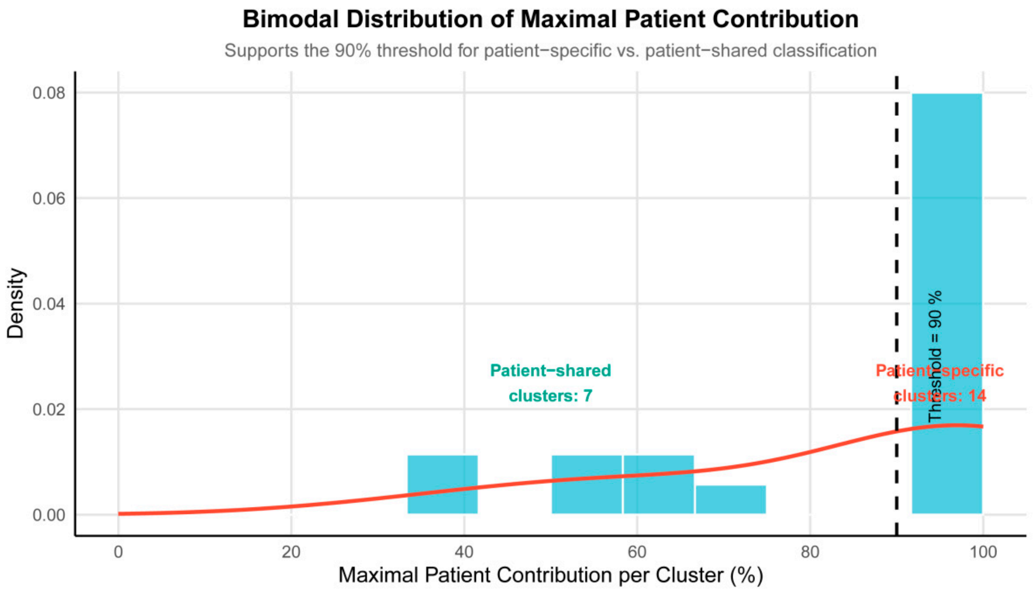

Supplementary Figure S2. Distribution of maximal patient contribution across epithelial subclusters.

The density distribution of maximal patient contribution per epithelial subcluster shows a distinct peak at ≥90%， indicating that a substantial fraction of subclusters are dominated by cells from individual patients. In contrast, subclusters with ≤60% contribution exhibit low density and broad dispersion. This bimodal pattern validates the use of a 90% threshold to distinguish patient-specific (≥90%) from patient-shared (<90%) subclusters.

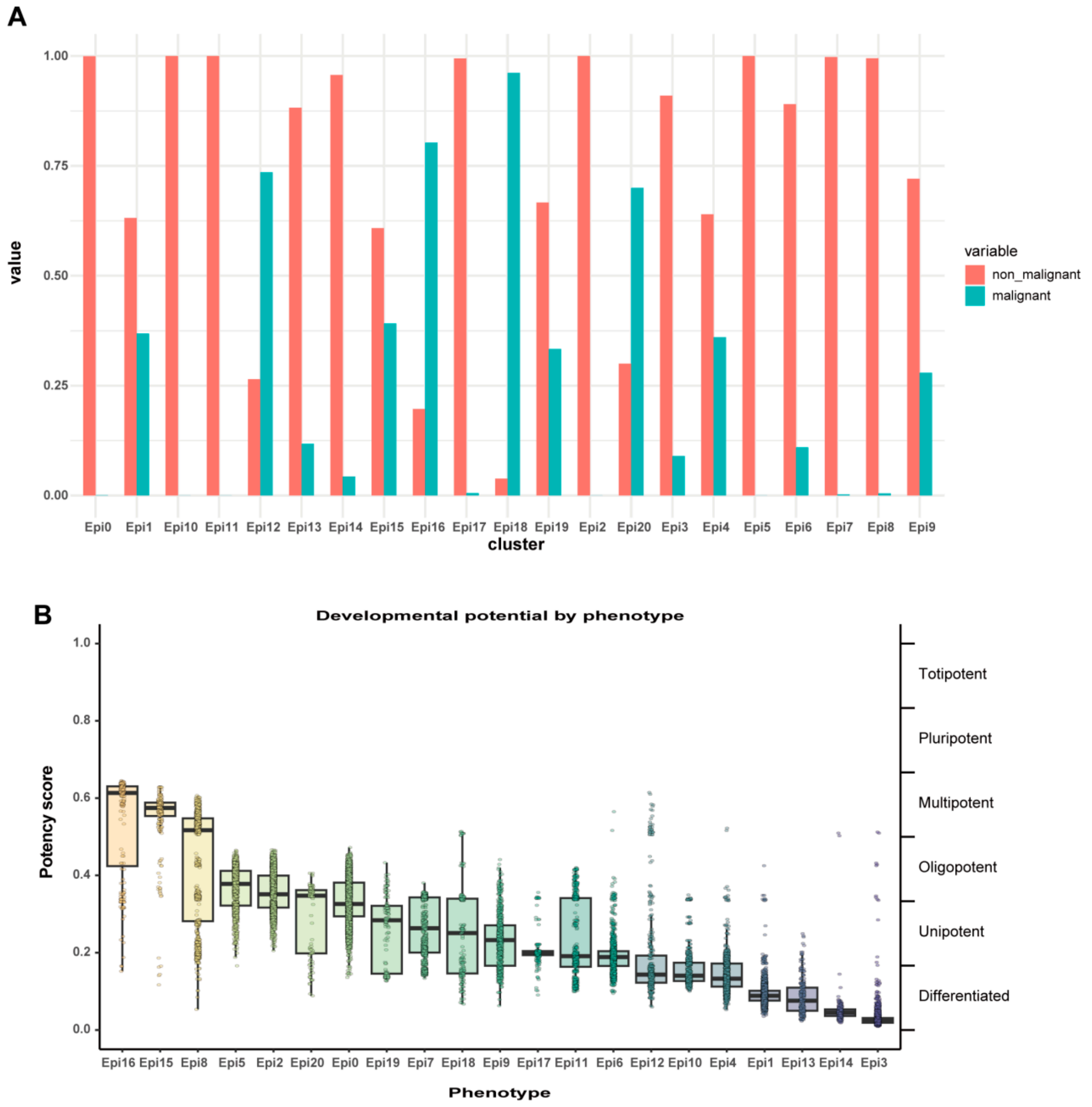

Supplementary Figure S3. Analysis of malignant epithelial cells

(A)Ratio of tumor to non-tumor cells in epithelial subpopulations; (B) Differentiation states: CytoTRACE2_Score (0 – 1, higher = more potential) and Potency classification.

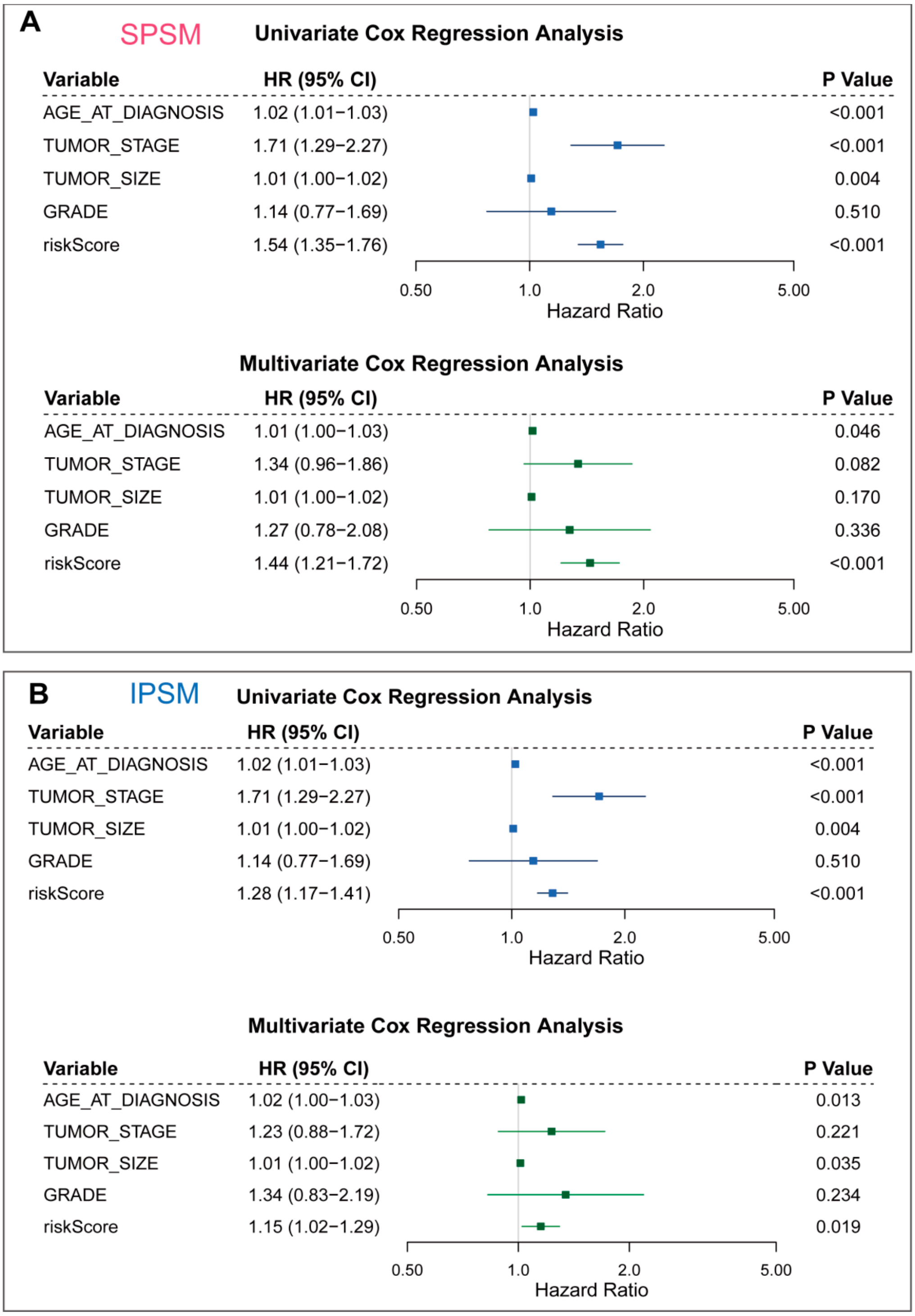

Supplementary Figure S4. Validation of the independent prognostic value of the risk model.

(A)Univariate and multivariate Cox regression analyses of the risk score for overall survival in the SPSM model;(B)Univariate and multivariate Cox regression analyses of the risk score for overall survival in the IPSM model.

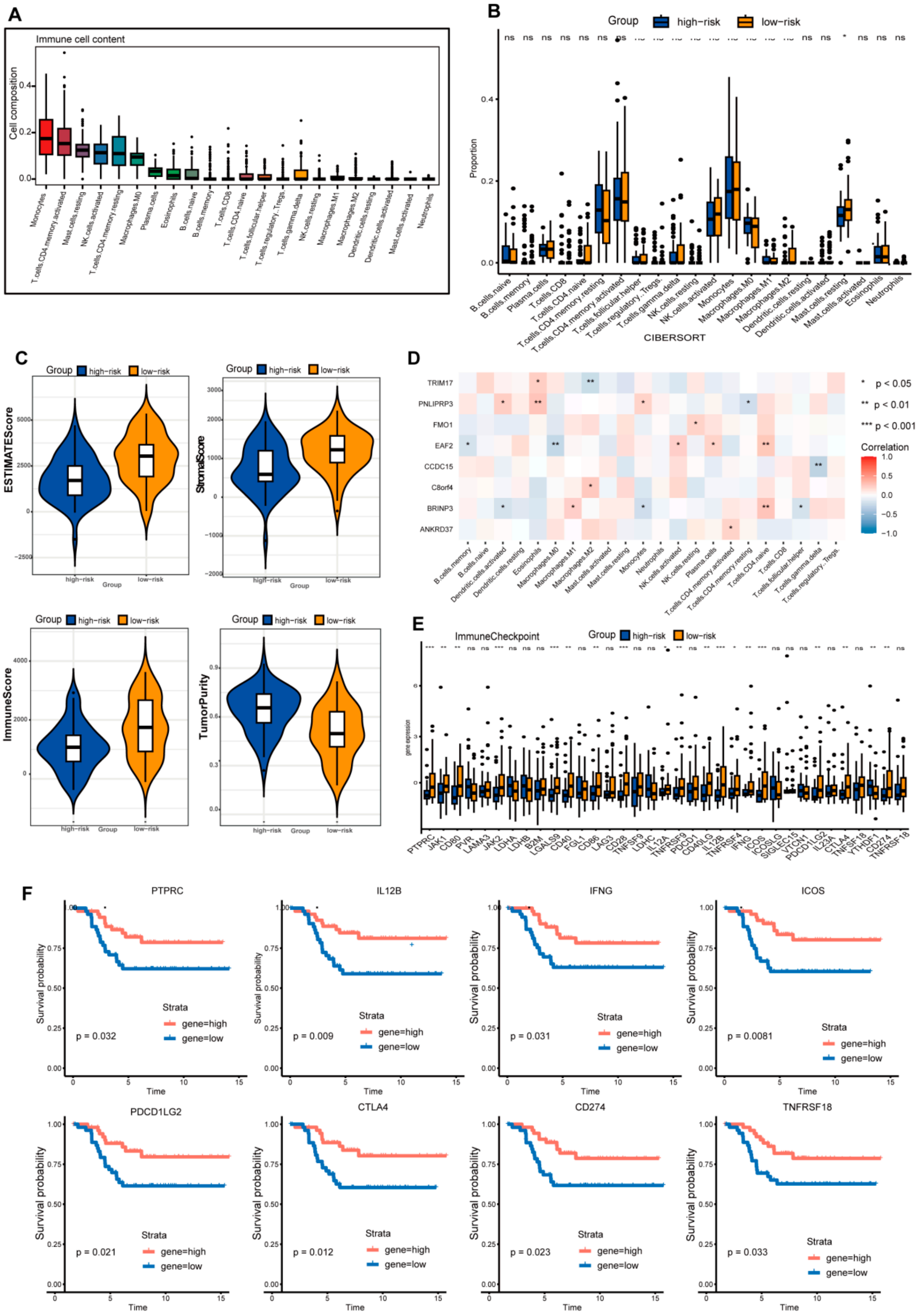

Supplementary Figure S5. Differences in immune-related characteristics between high- and low-risk score groups based on the IPSM model

(A) Immune infiltration profiles of 22 immune cell types in TNBC samples; (B) Differences in immune cell infiltration between the high-risk score group and the

low-risk score group; (C) Comparative analysis of stromal cell and immune cell score differences between high-risk and low-risk groups; (D) Correlation between risk score genes and immune cell infiltration; (E) Analysis of immune checkpoint expression levels between high-risk and low-risk groups, with $P < 0.05$, $P < 0.01$, and$P < 0.001$; (F) Immune checkpoints associated with TNBC prognosis.
